# Perinatal Outcomes In Monozygotic Pregnancies Resulting From Assisted Reproductive Technology Procedures: A Single-Center 6-Year Experience Based On A Large Cohort Of Pregnancies

**DOI:** 10.5935/1518-0557.20220014

**Published:** 2023

**Authors:** Nur Dokuzeylul Gungor, Tugba Gurbuz

**Affiliations:** 1 Associate Professor, Bahcesehir University Göztepe, Medical Park Hospital, IVF Unit, Istanbul, Turkey; 2 Associate Professor, Nisantasi University, Istanbul, Turkey

**Keywords:** Infertility, monozygotic twin, *in vitro* fertilization, miscarriage, maternal risk

## Abstract

**Objective:**

Monozygotic twin (MZT) pregnancies increase the risk of maternal and infant mortality and include many complications. The present study describes our assisted reproductive technology (ART) procedures from the viewpoint of perinatal outcomes in MZT pregnancies.

**Methods:**

In this retrospective clinical cross-sectional study, 1159 *in vitro* fertilization (IVF) cycles performed between October 2014 and December 2019 were reviewed and perinatal outcomes and general clinical conditions analyzed.

**Results:**

Sixteen MZT pregnancies were observed, resulting in an incidence of 1.38%. The MZT pregnancy incidence for patients aged ≤35 and >35 years were 0.2% and 1.1%, respectively. Eight MZT pregnancies resulted in live births, while five ended in miscarriage. A significant positive correlation was found between the number of attempts and the age of female (r:0.674; *p*=0.004) and male (r:0.657; *p*=0.006) partners. Cumulus-Oocytes Complexes (COC) (r:0.635; *p*=0.008), Metaphase II Oocyte (MIIO) (r:0.627; *p*=0.009), Pronucleus Oocyte (PO) (r:0.585; *p*=0.017) were correlated with serum AMH levels. The number of MZT was positively correlated with male partner age (r:0.527; *p*=0.036) and negatively correlated with embryo transfer day (ETd) (r:-0.548; *p*=0.028).

**Conclusions:**

The incidence of MZT pregnancies observed in this study was similar to the incidence reported in the literature, although risk was more pronounced among women aged >35 years. Due to potential risks for mothers and fetuses, MZT pregnancies may become a problem as the number of individuals seeking IVF continues to increase.

## INTRODUCTION

Generally regarded as the failure to perform reproductive function, infertility is a global health problem (Sarac & Koc, 2018). Although infertility treatments vary depending on the source of the problem, age, ovarian difficulty in egg release, drinking or smoking, intense physical and mental stress are also known to cause it (Eker *et al*., 2019; Inal *et al*., 2018). Assisted reproductive technology (ART) encompasses infertility treatments in which the fertilization of eggs and sperm occurs outside the human body (Aboulghar *et al*., 2020; Ballesta-Castillejos *et al*., 2019). It includes a roster of techniques such as pre-implantation genetic screening, embryo cultures, intracytoplasmic sperm injection, fresh or frozen embryo transfer, and *in vitro* fertilization (IVF) with donor oocytes (Cakiroglu & Tiras, 2020).

ART has been practiced for nearly 40 years, and more than a million babies have been born as a result of IVF. The use and effectiveness of IVF have increased over time (Calik & Bulut, 2020). Frozen embryo transfer and intracytoplasmic sperm injection have played a significant role in the achievement of strong reproductive results (Gurbuz *et al*., 2020). Multiple pregnancies account for more than 30% of all pregnancies resulting from ART, and more than half of newborns are the product of multiple pregnancies (Jančar *et al*., 2018). Prematurity and other complications may occur in these cases and, consequently, babies born from multiple pregnancies have higher rates of hospitalization and mortality compared to infants born from singleton pregnancies (Cavoretto *et al*., 2018).

The frequency of anomalies in multiple pregnancies is higher than in singleton pregnancies (Elias *et al*., 2020). Except for some problems that can be treated prenatally, non-vital anomalies cause new problems for both obstetricians and parents in multiple pregnancies (Dobrosavljevic *et al*., 2019). Although twin births account for 2% of all births, 8% of perinatal deaths occur in multiple pregnancy cases (Fernandes *et al*., 2016). Mortality is four times higher in multiple pregnancies than in singleton pregnancies and perinatal mortality is 19-32% (Zegers-Hochschild *et al*., 2017). Specific problems associated with high mortality in multiple pregnancies are common in monochorionic twins (Fineman *et al*., 2019). Monochorionic placentation increases the frequency of congenital anomalies in monozygotic twin (MZT) pregnancies. Compression-related deformations and lower extremity deformities occurring in the last stages of pregnancy and related to intrauterine posture position are not uncommon in multiple pregnancies (Arıoğlu Aydın *et al*., 2016).

Since MZT pregnancies increase the risk of maternal and infant mortality and may entail many complications, sharing data from ART treatments is important to ensure the adoption of strict treatment standards and to monitor perinatal risks. The present study describes our assisted reproductive technology (ART) procedures from the viewpoint of perinatal outcomes in MZT pregnancies.

## MATERIALS AND METHODS

### Study Design

We retrospectively evaluated 1159 cycles included in the ICSI program of the Department of Obstetrics and Gynecology, Faculty of Medicine, between October 2014 and December 2019. We analyzed the perinatal outcomes and general clinical conditions of all monozygotic pregnancies. This retrospective clinical cross-sectional study was conducted following approval by the Bahcesehir University Ethics Committee (17.07.2020-0003). The experiment protocol was developed in accordance with national, international, and institutional guidelines for studies involving human beings and complied with the principles of the Declaration of Helsinki. Informed consent was obtained from all subjects and the data collected included basic patient clinical condition, delivery results, type of conception, and pregnancy outcomes. Patient data were obtained from the retrospective examination of test results and inpatient treatment charts. The cases included in the study were evaluated in terms of average age of pregnant women, average gestational week, delivery type, birth weight, presence of EMR, gender, cesarean indications, miscarriage, fetal problems, fetal reduction, discordance between fetuses, pregnancy anemia, blood transfusion to mother, cerclage, abortus imminens, emesis gravidarum, BMI.

### Inclusion & Exclusion Criteria

Women were identified through the use of an electronic database. The result of all clinical and ultrasound examinations were considered and only MZT pregnancies reaching week 16 of gestation were included. Other types of pregnancy or related conditions were excluded from the present study. In detailed ultrasound analysis, pregnancies were accepted as monochorionic based on the presence of placenta and the absence of the twin peak mark known as the “lambda sign”. An experienced team of gynecologists specialized in obstetric sonography and IVF performed the clinical assessments and ultrasound examinations of the patients.

### Statistical Analysis

The SPSS program for Windows (v16.0, SPSS Inc. Illinois,USA) was used to evaluate the study results. Descriptive statistics outputs were given as mean ± standard deviation for continuous numerical variables or median (minimum-maximum) and percentage for categorical variables. Comparative analysis was done with the Mann-Whitney U test for continuous data and the χ2 test for categorical data. *p*<0.05 was considered statistically significant in data assessment.

## RESULTS

Sixteen of the 1159 IVF cycles resulted in MZT pregnancy, yielding an incidence of 1.38%. The incidence of MZT pregnancy in individuals aged ≤35 and >35 years were 0.2% and 1.1%, respectively. As seen in Table 1, the main reason for infertility was male-related factors (n:7). Other reasons were endometriosis (n:3), Duchenne muscular dystrophy (n:2), tubal (n:1), and unexplained (n:3). Female patients were aged 29.8±3.4 years and males were aged 33.7±3.2 years on average. The mean BMI was 24.2±2.3 kg/m^2^. The mean number of attempts was 2.1±2.2 and the mean AMH level was 2.4±1.7.

**Table 1:** Patient demographic data.

Female Age	Male Age	BMI	Trial	AMH	Reason
26	30	28	0	3	Genetic-DMD
36	39	22	8	1	Male-Related
28	31	23	0	0.7	Unexplained Infertility
29	34	23	2	1	Unexplained Infertility
25	35	24	3	0.7	Male-Related
27	36	24	0	5	Male-Related
25	27	22	2	2	Male-Related
31	34	27	4	1	Tubal
27	30	25	0	4	Genetic-DMD
31	33	22	2	5	Endometriosis
31	38	24	3	1	Unexplained Infertility
30	32	23	0	1	Endometriosis
32	35	26	2	1	Male-Related
36	38	27	5	5	Male-Related
30	33	20	0	3	Endometriosis
32	34	27	2	4	Male-Related

**Abbreviations.** DMD: Duchenne Muscular Dystrophy

Eight MZT pregnancies resulted in live births. Two of them were triplet pregnancies (25%). Five MZT pregnancies resulted in miscarriage. Two of them were triplet pregnancies (40%). Three MZT pregnancies, which were twin pregnancy, resulted in partial abortion. Table 2 shows ART cycle characteristics.

**Table 2:** Patient outcomes with oocyte and trigger details Trigger Agent.

Trigger Agent	Outcome	COC	MII	PO	ETD	ETF
Ovitrelle	Live Birth-Twin	12	10	10	5	1
Ovitrelle	Live Birth-Twin	8	7	7	5	2
Ovitrelle	Live Birth-Twin	3	3	3	5	1
Ovitrelle	Live Birth-Three	3	3	3	4	2
Ovitrelle	Live Birth-Three	3	3	3	3	2
Ovitrelle	23w Two Baby Ex	5	4	3	5	1
Ovitrelle	Live Birth-One	8	8	6	5	2
Ovitrelle	Live Birth-Twin	9	7	6	5	2
Ovitrelle	Live Birth-Twin	9	8	8	5	1
Lucrin	Live Birth-One	14	13	12	5	2
Dual	22w Three Abort	5	4	4	5	2
Ovitrelle	11w Twin Missed	7	6	6	5	1
Lucrin	15w Abort-Three	10	8	8	5	2
Lucrin	Live Birth-One	15	12	11	5	2
Lucrin	Live Birth	11	9	8	5	1
Lucrin	Spontaneous Abortion	11	8	8	5	2

**Abbreviations.** COC: Cumulus-Oocytes Complexes, MII: Metaphase II Oocytes, PO: Pronucleus Oocytes, ETD: Embryo Transfer Day, ETF: Frequency of Embryo Transfer

A significant positive correlation was found between the number of attempts and the age of the female (r:0.674; *p*=0.004) and male (r:0.657; *p*=0.006) partners. Similarly, Cumulus-Oocytes Complexes (COC) (r:0.635; *p*=0.008), Metaphase II Oocyte (MIIO) (r:0.627; *p*=0.009), Pronucleus Oocyte (PO) (r:0.585; *p*=0.017) were correlated with serum AMH levels. Number of MZT was positively correlated with the male age (r:0.527; *p*=0.036) and negatively correlated with embryo transfer day (ETd) (r:-0.548; *p*=0.028).

## DISCUSSION

The need to develop their careers has led many couples to delay their plans of having children. A growing number of couples seek ART treatments at a more advanced age. Although a rare event, previous studies have reported that the incidence of MZT has increased more than four times as a result of IVF procedures, with an increased risk of obstetric complications and poor pregnancy outcomes (Delrieu *et al*., 2012; Franasiak *et al*., 2015; Gee *et al*., 2014). For these reasons, MZT pregnancies may become a problem as the number of individuals seeking IVF continues to increase. In that term, the present study will contribute to the MZT numbers resulting from the application of IVF in our country and the perinatal clinical outcomes of MZT.

Despite the troublesome effects of MZT pregnancies, in many clinics there still is a preference for transferring multiple embryos to increase the chance of pregnancy (Tummers *et al*., 2003). Decreased fertility possibly occurs mainly due to oocyte aging rather than poor endometrial receptivity. Older women produce fewer oocytes and have lower implantation rates, suggesting that follicles are less responsive to exogenous hormones, which ultimately leads to the retrieval of fewer high-quality oocytes (Chuang *et al*., 2003). Sotiroska *et al*. (2015) found higher pregnancy rates in individuals receiving ET on the 5^th^ day. However, they observed a strong decrease in delivery/pregnancy rates in older individuals (age >36 years) compared to younger ones. This suggests that older women have a lower chance of conceiving through assisted reproductive technology, despite having low basal FSH levels. In the present study, the number of MZT was positively correlated with male age and negatively correlated with embryo transfer day. A significant positive correlation was found between the number of attempts and age of female and male partners.

Recent studies have looked into various aspects of IVF procedures, parent age, hormone levels, and anatomical problems previously associated with the incidence of MZT pregnancies. According to Knopman *et al*. (2014), the likelihood of having an MZT pregnancy was linked to the possibly superior reproductive potential of younger individuals reflected in the supply of healthier oocytes. Although logical, there is still a lot of uncertainty in this explanation and more data is needed. Embryos derived from younger oocytes are transferred in the blastocyst stage of the advanced levels. This because age factor may not be an independent risk factor for MZT, but rather a representative of blastocyst transfer (Knopman *et al*., 2014). In the study by Sills *et al*., the incidence of MZT pregnancies was 1.3%, a proportion comparable to reported results and three times higher than the incidence of naturally conceived MZT pregnancies, described as 0.4% (Sills *et al*., 2000). Vega *et al*. (2018), in the largest study of IVF cycles evaluating multiple pregnancies, dizygotic, and discordant twinning rates, reported a rate of MZT pregnancies in women aged <35 years of 1.7%. Osianlis *et al*. (2014) analyzed a large single-institution database and determined that cycles carried a 2.3% risk of multiple pregnancies. In our study with 1159 IVF cycles, sixteen resulted in MZT pregnancies, an incidence of 1.38%. The incidence of MZT pregnancies in women aged ≤35 and >35 were 0.2% and 1.1%, respectively.

As a result, high-risk results in terms of maternal and infant health can occur in IVF. Shevell *et al*. (2005) detected an increased abnormal placentation rate in IVF and assumed that it may cause complications during pregnancy. Romundstad *et al*. (2006) found that placenta previa occurs six times more frequently in singleton pregnancies after ART procedures. Zhu *et al*. (2016) reported abnormal placental cord placement in women who gave birth after ART procedures versus matched controls with spontaneous pregnancies. Considering these studies, it has been suggested that MZT pregnancy may cause poor maternal outcomes and stillbirths resulting from inadequate or abnormal placental development. In our study, we observed that eight MZT pregnancies resulted in live births. Five MZT pregnancies resulted in miscarriage. Three MZT pregnancies - twin pregnancies - resulted in partial abortion. These MZT pregnancy outcomes corroborate the existence of increased maternal risk described in the literature.

The effect of low AMH levels on oocyte quality is known. However, the possibility that the association between AMH and the presence of oocyte defects may result from decreased granulose secretion in poor quality oocytes remains an important issue that continues to be investigated. AMH levels may influence the determination of the dominant follicle through the inhibitory effects of AMH during the primary follicle collection process from the primordial pool (Durlinger *et al*., 2001) and regulation of FSH sensitivity in ovarian tissue (Kevenaar *et al*., 2007). Borges *et al*. (2017) and Fanchin *et al*. (2007) examined whether follicular AMH production was positively associated with oocyte and embryo development and demonstrated its significant effect. Ebner *et al*. (2006) showed that AMH levels are directly and strongly associated with oocyte quality. Loh & Maheshwari (2011) explained that AMH did not predict the likelihood of pregnancy. La Marca *et al*. (2011) reported that extremely low levels of AMH were associated with inability to conceive. Lamazou *et al*. (2011) found reasonable pregnancy rates following extremely low serum AMH levels. Borges *et al*. (2017) showed that the probability of pregnancy and the number of embryos obtained, high-quality embryos, and the number of embryos transferred were positively correlated with AMH level. Similarly, in the present study, we found that cumulus-oocytes complexes, metaphase-II oocytes, and pronucleus oocytes were strongly correlated with serum AMH levels.

The present study has its limitations. Not all patient data related to risk factors for MZT pregnancies resulting from ART procedures were identified in this retrospective study. Another point is the fact that the number of women who achieve pregnancy with the aid of IVF or through natural conception may vary according to demographic variables potentially associated with MZT pregnancy. Since being a young female is directly connected with having MZT pregnancy after ART, we cannot exclude the possibility of having underestimated the risk. This limitation is generally seen in clinical observational analysis in different medical fields. In the present study, however, it should be noted that although some confounders cannot be ruled out, the magnitude of the risk tends to support the existence of a causal effect.

## CONCLUSION

The incidence of MZT pregnancy among the IVF patients included in our study was similar to the incidence reported in the literature, although risk was more pronounced in women aged >35 years. A strong positive correlation was found between the number of attempts and the age of the female and male partners. The number of MZT pregnancies was positively correlated with male age and negatively correlated with embryo transfer day. As a result, MZT pregnancies may become a problem as the number of individuals seeking IVF continues to increase due to its potential risks for both maternal and fetus.

**Ethics approval and consent to participate:** The study was approved by the Ethics Committee at Bahcesehir Univercity with the date and number: 17.07.2020-0003. Informed consent was obtained from all subjects.

**Consent for publication:** Approved by Bahcesehir University (17 July 2020).
